# Overweight and obesity significantly reduce pregnancy, implantation, and live birth rates in women undergoing *In Vitro* Fertilization procedures

**DOI:** 10.5935/1518-0557.20200105

**Published:** 2021

**Authors:** Javier García-Ferreyra, Jorge Carpio, Milton Zambrano, Pedro Valdivieso-Mejía, Pedro Valdivieso-Rivera

**Affiliations:** 1 Laboratory of Assisted Reproduction. Alcívar Hospital, Guayaquil, Ecuador

**Keywords:** Body mass index, IVF, pregnancy, implantation, live birth, ART

## Abstract

**Objective:**

The objective of this study was to evaluate the effects of overweight and obesity on fertility outcomes in IVF procedures.

**Methods:**

This was a retrospective and nonrandomized study that included 191 IVF/ICSI cycles using non-donor oocytes performed between July 2016 and December 2018 that were allocated according to Body Mass Index (BMI) in three groups: Normal group: 18.5-24.9 (n=67 women), Overweight group: 25.0-29.9 (n=86 women) and Obesity group: ≥30.0 (n=38 women). We compared fertilization rates, embryo quality at day 3, development and quality of blastocyst, pregnancy rates, implantation rates, and live birth rates.

**Results:**

Patients from all groups had similar stimulation days, but those women with overweight and obesity used more hormones compared to women with normal weight (*p*<0.05). Fertilization rates, zygotes that underwent cleavage and good-quality embryos at Day 3 were similar between the three evaluated groups. The groups of overweight and obesity had embryos at Day 3 with significantly less cells, compared to those from the normal group (*p*<0.05). The blastocyst development rate was significantly lower in women with overweight and obesity compared to women with normal BMI (*p*<0.05); but, the percentages of good blastocysts were similar in all studied patients. Pregnancy, implantation and live birth rates were significantly lower in the group of women with overweight and obesity, compared to those women with normal weight (*p*<0.05). Obese women had significantly more miscarriages compared to those in the other groups (*p*<0.05).

**Conclusions:**

Our data shows that an increased BMI affects embryo development and significantly reduces the pregnancy, implantation and live birth rates.

## INTRODUCTION

Overweight and obesity (defined as a body mass index (BMI) of 25-29.9 kg/m^2^ and ≥30 kg/m^2^, respectively), have become an epidemic and a major public health problem worldwide. Between 1980 and 2013, there was an increase in BMI above the normal level of 25 kg/m^2^, from 28.8 to 36.9% in men and 29.8 to 38% in women (Ng *et al*., 2014), and in 2016, the World Health Organization (WHO) stated that of the world’s adult population, 39% was overweight and 13% was obese; and of these, 40% were women with overweight and 13% women with obesity (WHO, 2016). Additionally, data from the National Health and Nutrition Examination Survey (NCHS) from USA reported that during 2015-2016, Hispanic women had a higher prevalence of obesity (50.6%) than non-Hispanic white (38.0%) and non-Hispanic Asian (14.8%) women, and this prevalence was similar to the one found in non-Hispanic white women (54.8%) (Hales *et al*., 2017).

It is evident that there are well-defined disparities in obesity rates between different ethnic and socioeconomic backgrounds, but one of the subpopulations that is mostly affected are women of reproductive age. Obese women have a threefold risk for infertility (Van Der Steeg *et al*., 2008; Brewer & Balen, 2010), decline in fecundability (Ramlau-Hansen *et al*., 2007; Wise *et al*., 2010), impaired ovulation, poor oocytes and embryo quality (Jungheim & Moley, 2010; Pandey *et al*., 2010), and higher cancellation rates (Fedorcsák *et al*., 2004; Luke *et al*., 2011a; Mackenna *et al*., 2017).

The principal effect of an abnormal BMI in women with overweight and obesity is increased levels of lipid storage in adipose tissues and other metabolic organs, which lead to cellular lipid toxicity, inflammation, oxidative stress, and development of metabolic dysfunctions that finally reduce their quality and quantity of life (Snider & Wood, 2019). In addition, overweight and obesity are associated with alterations to the hypothalamic-pituitary-gonadal-axis leading to menstrual dysfunction, anovulation, uterine bleeding, endometrial pathology, and increased risk of poor obstetrical outcomes, such as preeclamsia, gestational diabetes, induction of labor, surgical delivery, postpartum hemorrhage, fetal macrosomia, and congenital anomalies (Linné, 2004; Catalano & Ehrenberg, 2006; Stothard *et al*., 2009; Aly *et al*., 2010; Kasum *et al*., 2017).

In recent years, there has been a substantial improvement in quality control and culture systems in ART worldwide that has brought about an increase in implantation and pregnancy rates in those patients undergoing IVF/ICSI cycles; however, it is yet not clear the effect of overweight and obesity on clinical outcomes. Although some authors have reported no negative effects of obesity on IVF outcomes (Dechaud *et al*., 2006; Maheshwari *et al*., 2007; Farhi *et al*., 2010; Mackenna *et al*., 2017; Ben-Haroush *et al*., 2018), others have showed an adverse effect of overweight and obesity on pregnancy rates (Fedoresák *et al*., 2004; Bellver *et al*., 2010; Luke *et al*., 2011a; Rittenberg *et al*., 2011; Moragianni *et al*., 2012; Kawwass *et al*., 2016; Luke, 2017; Sampo *et al*., 2017), implantation rates (Bellver *et al*., 2010; Moragianni *et al*., 2012), miscarriages rates (Veleva *et al*., 2008; Rittenberg *et al*., 2011; Kawwass *et al*., 2016), and live birth rates (Bellver *et al*., 2010; Luke *et al*., 2011b; Rittenberg *et al*., 2011; Moragianni *et al*., 2012; Petersen *et al*., 2013; Kawwass *et al*., 2016). A systematic review and meta-analysis of 33 studies and 47,967 IVF/ICSI procedures revealed that overweight or obese women had significantly lower clinical pregnancy and live birth rates, and significantly higher miscarriage rates (Rittenberg *et al*., 2011). Similar results were reported by Maheshwari *et al*. (2007), Vural *et al*. (2015), Kawwass *et al*. (2016), and Provost *et al*. (2016). Additionally, a meta-analysis showed that overweight and obese women have a 10% lower live birth rate when compared to their normal counterparts when undergoing an IVF procedure (Ku *et al*., 2006).

The aim of our study was to investigate the relationship between overweight and obesity with clinical outcomes and embryo development in fresh IVF/ICSI cycles.

## MATERIALS AND METHODS

### Study design

This is a retrospective and nonrandomized study based on data analysis from 191 IVF/ICSI cycles with fresh embryo transfer, performed between July 2016 and December 2018 at the Laboratory of Assisted Reproduction of Alcivar Hospital (Guayaquil, Ecuador). BMI was calculated by dividing body mass (weight in kilograms) by the square of body height in meters. All cycles were allocated according to BMI in three groups: normal group: 18.5-24.9 kg/m^2^ (*n*=67 women), overweight group: 25.0-29.9 kg/m^2^ (*n*=86 women) and obesity group: ≥30.0 kg/m^2^ (*n*=38 women). We included those patients admitted to autologous IVF and ICSI with one or more mature oocytes recovered and one or more viable embryo obtained. We did not include cases of azoospermia and egg donation.

All participants and their husbands signed informed consents and the Institutional Review Board and the corresponding Ethics Committee from the Alcivar Hospital (Guayaquil, Ecuador) approved the study.

### Controlled ovarian stimulation and oocyte collection

We stimulated the menstrual cycles of patients using recombinant follicle-stimulating hormone (rFSH) (Gonal^®^, Merck Serono laboratories, Ecuador), according to established stimulation protocols, on day 2 of the menstrual cycle until at least two follicles of ~18 mm in diameter were reached. Oocytes were collected 36h after recombinant human chorionic gonadotropin administration (Ovidrel^®^, Merck Sereno laboratories, Ecuador) by transvaginal ultrasound ovum pick-up, with the patient under general anesthesia with 200 mg of Propofol iv (Diprivan^®^ 1% P/V; AstraZeneca Laboratories, UK). During the follicular aspiration procedure, the oocytes were recovered in the Global^®^-HEPES-buffered medium (LifeGlobal) supplemented with 10% vol/vol Serum Substitute Supplement (SSS; Irvine Scientific). After retrieval, cumulus-oocyte complexes were trimmed of excess cumulus cells using sterile needles and cultured in ~150 µl drops of Global^®^-Fertilization medium (LifeGlobal), plus 10% SSS under oil at 37ºC in an atmosphere containing 6% CO_2_, 5% O_2_ and 89% N_2_ for 5 hours before the IVF/ICSI procedure.

### Insemination, fertilization and embryo culture

The recovered oocytes were assessed for their nuclear maturity, and only metaphase II oocytes were submitted to IVF/ICSI. We inseminated the patients with 50,000-100,000 motile spermatozoa in ~150 µl drops of Global^®^-Fertilization medium + 10% SSS, where 1 to 5 oocytes were placed. In the ICSI procedures, all collected oocytes were denuded enzymatically off cumulus cells with hyaluronidase (80 IU/ml; LifeGlobal), and injected following routine laboratory procedures.

We confirmed normal fertilization 16-18 hours after insemination/injection by the presence of two pronuclei (day 1). The zygotes were individually cultured under mineral oil, in 20-µl droplets of Global^®^ medium (LifeGlobal), supplemented with 10% vol/vol SSS from day 1 to day 3, in which the embryos were moved to fresh Global^®^ medium and cultured 2 days more up to blastocyst stage. On day 3, the embryos were evaluated for cell number, fragmentation and multinucleation, and on day 5, for development of blastocyst and expansion. Good quality day-3 embryos were defined as those with 6-8 cells and ≤10% fragmentation. Good quality blastocysts were defined as having an inner cell mass (ICM) and trophectoderm type A or B (Gardner & Schoolcraft, 1999). The ICM score was evaluated as follows: type A = compact area, many cells present; type B = cells are loosely grouped. The trophectoderm was scored as follows: type A = many cells forming a tight epithelial network of cells; type B = few cells forming a loose network of cells.

### Seminal samples

On the day of the IVF/ICSI procedure, all patients’ partners collected the semen samples by masturbation in aseptic conditions, into sterile cups after 2 days of sexual abstinence. Concentration, progressive motility and morphology from spermatozoa were assessed after semen liquefaction for 30 minutes at room temperature, according to World Health Organization criteria (WHO, 2010). Motile spermatozoa were separated from the seminal plasma by centrifugation at 300 X g for 10 minutes through 1.0 ml 95% and 45% Isolate gradients (Irvine Scientific, USA). The pellet was washed once by centrifugation for 5 minutes, and was resuspended in 0.3 ml of Global Fertilization medium + 10% SSS for IVF/ICSI.

### Embryo transfer

Embryos were transferred on day 5 in all patients using a Rocket Bulb Tip embryo transfer catheter (Rocket Medical, USA) that had been previously washed with culture medium. The catheter was completely filled with culture medium and the blastocysts filled the bottom 10 µl of the catheter. All transfers were performed according to the methods previously described by Mansour (2005). The blastocysts that were not transferred were cryopreserved or discarded according to their morphology.

### Pregnancy determinations

Biochemical pregnancy was assessed 14 days after the embryo transfer by measuring the Human Chorionic Gonadotropin beta subunit (hCG-b) in the blood. The clinical pregnancy rate (PR) was determined by transvaginal ultrasonography to detect gestational sacs and fetal heartbeats at approximately 21 and 28 days after transfer, respectively. The implantation rate (IR) was determined by dividing the number of gestational sacs by the number of embryos transferred. Live birth rate (LBR) was determined by dividing the number of cycles that lead to live birth by the total number of transferred cycles.

### Statistical Analysis

The data was statistically analyzed using the χ^2^ test, Student’s t-test, and multivariate analysis as appropriate, and differences were considered to be significant at *p<*0.05. All statistical analyses were carried out using the statistic package Stata 10 (StataCorp, College Station, TX, USA).

## RESULTS

Our study analyzed 191 fresh IVF/ICSI cycles using non-donor oocytes, of which 67 women had normal weight (35.1%), 86 were overweight (45.0%) and 38 were obese (19.9%). The age of the patients and their partners were similar between the evaluated groups (Table 1). No difference was found in duration of ovarian stimulation according to the BMI group; but overweight and obese women used higher doses of rFSH, compared to the normal women group (*p<0.05*) (Table 1). In regards to seminal samples of patients’ husbands, parameters of volume, sperm concentration, progressive motility and sperm morphology were similar between the three groups in the study (Table 2).

**Table 1:** Characteristics of patients according to their BMI.

	Normal	Overweight	Obesity
Cycles	67	86	38
BMI (Mean±SD)	22.8±1.61	27.4±1.28*	33.5±3.76*,**
Female age (years) (Mean±SD)	33.9±3.48	33.7±3.86	33.5±3.47
Male age (years) (Mean±SD)	35.9±5.78	37.8±7.45	39.5±8.32
Stimulation days (Mean±SD)	9.0±1.15	9.2±1.18	9.1±1.01
rFSH (U/L) (Mean±SD)	1683.2±456.42	1882.1±422.95*	2143.8±337.46*

* *p*<0.05 in relation to the normal group

** *p*<0.05 in relation to the overweight group

**Table 2:** Seminal characteristics in the evaluated groups.

	Normal	Overweight	Obesity
Semen volume (ml) (Mean ± SD)	2.5 ± 1.23	2.4 ± 1.09	2.5 ± 1.02
Sperm concentration (x 106/ml)	56.8 ± 33.96	56.5 ± 29.59	58.7 ± 25.58
Progressive motility (%)	24.2 ± 11.99	24.9 ± 12.73	27.6 ± 11.18
Sperm morphology (%)	8.4 ± 3.21	8.7 ± 4.59	8.3 ± 7.07

*p*: not significant (NS)

Table 3 summarizes the laboratory results in the study groups. A total of 455, 587 and 288 oocytes were inseminated from normal weight, overweight and obesity groups, respectively. Fertilization rate (2PN) (87.7%, 85.3% and 84.0%), percentages of zygotes that underwent cleavage (97.2%, 99.2% and 98.8%), and good-quality embryos at day 3 (84.0%, 82.3% and 79.9%) were similar in the three evaluated groups (*p=*not significant). However, embryos from overweight and obese women had significantly lower cell numbers compared to those embryos from normal-weight women (6.7±1.73 and 6.8±1.04 versus 7.3±0.79 cell/embryo) (*p<0.05*). During extended embryo culture there was a significantly negative effect of BMI above normal values on blastocyst development rate (*p<0.05*). The quality and expansion degree of blastocysts were no affected by BMI values.

**Table 3:** Comparison of laboratory results between the three evaluated groups.

BMI	Normal	Overweight	Obesity
No. total oocytes	586	771	338
No. total inseminated oocytes	455	587	288
No. total fertilized oocytes (2PN) (%)	399 (87.7)	501 (85.3)	242 (84.0)
No. total cleaved embryo at Day 3 (%)	388 (97.2)	497 (99.2)	239 (98.8)
No. cell/embryo at Day 3 (Mean ± SD)	7.3±0.79	6.7±1.73 *	6.8±1.04 *
Good-quality embryos at Day 3 (%)	84.0	82.3	79.9
Blastocyst formation rate (%)	48.2	40.0 *	40.1 *
Good-quality blastocysts (%)	92.9	91.5	88.7
Full blastocyst (%)	13.4	11.6	18.6
Expanded blastocyst (%)	80.2	83.4	78.3
Hatching blastocyst (%)	6.4	5.0	3.1

* *p*<0.05 in relation to normal group

Table 4 shows the clinical outcomes. The mean number of blastocysts available from normal, overweight and obese women were similar (2.92±2.02, 2.52±1.71 and 2.24±2.45, respectively). At day 5, we transferred comparable numbers of embryo (1.84±0.37, 1.84±0.38 and 1.86±0.31) to women of normal, overweight and obese groups, respectively (*p=*not significant). The clinical PR (41.9 and 39.5% versus 67.2%), IR (31.8 and 28.2% versus 53.7%) and LBR per transfer (52.3 and 39.5 versus 89.6%) were significantly lower in those women with overweight and obesity, when compared to those with normal weight (*p<0.05*). Obese women had a significantly higher miscarriage rate compared to the other groups evaluated (*p<0.05*). We found one gestational sac was in 24 (53.3%) patients in the normal group, 24 (66.7%) patients of overweight group, and 12 (80.0%) patients of obesity group, respectively. There were two gestational sacs in 21 (46.7%) patients of the normal group, in 12 (33.3%) patients in the overweight group, and in 3 (20.0%) patients in the obesity group, respectively. Women with normal BMI had significantly more twin pregnancies when compared to those women from other groups (*p<0.05*).

**Table 4:** Clinical outcomes in the normal, overweight and the obesity groups.

BMI	Normal	Overweight	Obesity
Cycles	67	86	38
No. blastocyst/patient (Mean ± SD)	2.92±2.02	2.52±1.71	2.24±2.45
No. of embryo transferred/patient (Mean ± SD)	1.84±0.37	1.84±0.38	1.86±0.31
Pregnancy rate (%)	67.2	41.9 *	39.5 *
Implantation rate (%)	53.7	31.8 *	28.2 *
Live birth rate (%)	89.6	52.3 *	39.5 *
Miscarriages (%)	2.2	2.7	6.7 *
Single pregnancy (%)	53.3	66.7	80.0
Twin pregnancy (%)	46.7 **	33.3	20.0

* *p*<0.05 in relation to normal group

** *p*<0.05 in relation to overweight and obesity groups

The multivariate analysis to assesses the possibility to achieve a pregnancy, controlling for BMI, patient age, stimulation days, rFSH doses, number of retrieved oocytes, fertilized oocytes, cleaved embryos at day 3, number or cells by embryo at day 3, blastocyst formation rate and number of embryos transferred as independent variables showed that only BMI was associated with the possibility of pregnancy success with a determination coefficient (*R2*) of 0.345 (Table 5).

**Table 5:** Multivariate logistic regression analysis for clinical pregnancy as a dependent variable.

Independent variable	ß	SE	*p*	Confidence interval at 95%	
BMI (%)	-0.04	0.02	0.017	-0.08	-0.01
Age of patient (years)	-0.04	0.02	0.072	-0.08	0.00
Stimulation days	-0.07	0.08	0.375	-0.23	0.09
rFSH doses (U/L)	0.01	0.01	0.555	-0.00	0.01
No. of retrieved oocytes	-0.04	0.03	0.162	-0.09	0.02
No. of inseminated oocytes	-0.08	0.05	0.131	-0.20	0.03
Fertilized oocytes (%)	0.16	0.18	0.369	-0.19	0.53
Cleaved embryos at Day 3 (%)	0.02	0.19	0.921	-0.36	0.40
No. cell/embryo at Day 3 (Mean±SD)	0.17	0.10	0.090	-0.03	0.37
Blastocyst formation rate (%)	-0.07	0.05	0.147	-0.17	0.03
No. embryo transferred	-0.04	0.36	0.273	-1.12	0.33

Dependent variable: Pregnancy Rate. ß = coefficient of regression, *SE* = standard error, *p* = probability, *N* = sample size. *R*^2^ = 0.345, P = 0.0047, *N* = 191.

## DISCUSSION

In the present study, we analyzed the effecst of BMI on clinical outcomes and perimplantation embryo development in women who underwent IVF/ICSI procedures with their own oocytes. Our data demonstrated that women with BMI above normal values (overweight and obesity groups) had significantly lower pregnancy, implantation and live birth rates during an IVF treatment. These results are very important because they show that overweight and obesity not only reduce the success of an IVF/ICSI procedure, but also delay the time-to-pregnancy and principally the possibility to have a term live born baby.

Obesity is a systemic and tissue-specific condition that can lead to chronic inflammation and oxidative stress, that produces an unregulated and persistent synthesis and secretion of chemokines and cytokines (Sapochnik *et al*., 2017; Varfolomeev & Vucic, 2018) by innate immune cells, and adipokines (e.g. leptin and lipocalin) from adipocytes (Garn *et al*., 2016; Kuroda & Sakaue, 2017). Cytokines and adipokines are released into the circulation, inducing inflammatory responses in several tissues, including the ovaries (Ouchi *et al*., 2011; Nteeba *et al*., 2013; Wang & Huang, 2015). Wu *et al*. (2006), Cohen-Fredarow *et al*. (2014), Field *et al*. (2014) and Dam *et al*. (2015) demonstrated that cytokines, chemokines and adipokines play essential roles in steroidogenesis, follicular growth, cumulus expansion after the LH surge, and ovulation; however, alterations in their patterns of synthesis and secretion impair meiotic and cytoplasmic maturation of the oocyte, reducing its developmental competence for fertilization and pre-implantation development. In relation to oxidative stress, obesity promotes the expression of the redox family of NADPH oxidases (NOX), which produced O_2_- (from organelles like mitochondria, endoplasmic reticulum and peroxisomes) that is converted to H_2_O_2_ by superoxide dismutase, which may freely move from organelles to the cytoplasm of the cell, thus increasing the expression of pro-inflammatory cytokines (Rimessi *et al*., 2016; Chong *et al*., 2017; Mailloux, 2018).

In this study, overweight and obese females had significant gonadotrophin consumption in comparison with normal weight women but did not find any significant differences in stimulation days. Similar results were reported by Maheshwari *et al*. (2007), Koatz & de Souza (2013), Ozekinci *et al*. (2015) and Ben-Haroush *et al*. (2018). These higher doses used in overweight and obese patients would be induced by an inhibitory effect of elevated leptin, which impairs ovarian responsiveness to gonadotrophin stimulation, requiring more medication or also to an increased clearance of the drugs by the excess of fat tissue (Souter *et al*., 2011).

In regards to laboratory results, there were no differences in fertilization rates, cleavage and good-quality embryo at day 3 across the BMI categories. However, patients with overweight and obesity had embryos at day 3 with significantly less cell/embryo and lower blastocyst formation rate when compared to patients with normal BMI, similar to what was previously reported by Comstock *et al*. (2015) and Leary *et al*. (2015). Excess free fatty acids in overweight and obese females affect the oocyte and its organelles through a lipotoxicity effect that will ultimately have a negative impact on the future embryo (Broughton & Moley, 2017). Obesity alters mitochondrial function in the oocyte, disrupting its architecture with fewer cristae, more vacuoles, and evidence of swelling. In addition, there are changes in their distribution with clumping throughout the ooplasm instead of a perinuclear localization (Grindler & Moley, 2013). Likewise, oocytes from women with obesity have showed disarrayed meiotic spindles with misaligned metaphase chromosomes (Machtinger *et al*., 2012), smaller size, minor oolemma diameter, and minor zona pellucida thickness than oocytes from women with normal BMI (Atzmon *et al*., 2017). In addition, human oocytes from overweight and obese patients have reduced glucose use and elevated endogenous triglyceride levels that significantly affect the oocyte competence, and its capacity of development to the blastocyst stage (Leary *et al*., 2015). These changes to the glucose and triglyceride metabolism are also reported in blastocysts from overweight and obese patients, and this would be one of the factors that reduces the proportion of blastocysts obtained during an IVF treatment in these patients (Comstock *et al*., 2015; Leary *et al*., 2015). Moreover, Yamada *et al*. (2013) evaluating the blastocyst formation rate according to the number of cells/embryos at day 3 in IVF/ICSI cycles reported that those embryos with <7 cells had a significantly reduced chance to reach the blastocyst stage, which was also reported during our study involving patients with BMI above normal values; and these minors percentages of blastocysts were independent of good-quality embryos at day 3 that was similar across the BMI categories. Herrero et al. (2013) and Leary et al. (2015) reported similar results in overweight/obese women whom embryos were less likely to reach the blastocyst stage; and those that did develop into blastocysts showed accelerated preimplantation development with fewer trophectoderm cells. Finally, it is important to highlight that our data demonstrate that the effect of overweight and obesity on oocyte competence and embryo development would start post-fertilization and not after day 3 of the cleavage stage, as per suggested by Comstock *et al*. (2015).

About clinical outcomes, all patients received similar numbers of fresh embryos in the blastocyst stage; however, there was a significant decline in PR, IR and LBR in overweight and obese patients compared to those with normal BMI. These data are similar to those reported by Vural *et al*. (2015), Provost *et al*. (2016), and Kudesia *et al*. (2018) concerning pregnancy rates; Moragianni *et al*. (2012) concerning implantation rates; and Bellver *et al*. (2010), Sifer *et al*. (2014), Kasum *et al*. (2017), Russo *et al*. (2017), and Supramaniam *et al*. (2018) concerning live birth rates. Likewise, our data shown that just those obese patients had significantly more miscarriages compared to other two evaluated groups, being these results similar to those reported in previous studies by Maheshwari *et al*. (2007) and Supramaniam *et al*. (2018). Additionally, these update results are in accordance with two big previous meta-analysis based on 47,967 and 682,532 cycles, which demonstrated that overweight and obesity have a significant deleterious effect on PR (Rittenberg *et al*., 2011) and LBR (Sermondade *et al*., 2019) following IVF.

Mechanisms such as impaired ovarian folliculogenesis, oocyte quality, embryonic development and endometrial receptivity have been proposed as an explanation to poor reproductive outcomes in overweight and obese women. Studies in human and animal models have shown that an altered function of the hypothalamic-pituitary axis associated with high insulin, androgen, estrogen, and leptin levels impair ovarian folliculogenesis, and therefore oocyte quality (Tortoriello *et al*., 2004; Jungheim & Moley, 2010). As explained above, oocyte and perimplantation embryo development are affected by obesity, through metabolic alterations to lipids, proteins and growth factors that lead to an increased oxidative stress and unbalance of certain free fatty acids that impair granulosa cells function (Lin *et al*., 2017), alignment of metaphase chromosomes in the meiotic spindles of oocytes (Machtinger *et al*., 2012), and glucose and triglycerides metabolism in blastocysts (Leary *et al*., 2015).

On the other hand, an altered endometrial receptivity could be responsible for decreased clinical outcomes in overweight/obese females such as we report in our study. Regarding controversial data whether an abnormal BMI affects the endometrium, Crosby *et al*. (2017) showed a significant positive correlation between female BMI and endometrial thickness (but not with the pregnancies), and such thickening is in the relative hyperestrogenic state due to excess aromatization in the adipose tissue. Likewise, it has been shown that obesity impairs endometrial decidualization, which is necessary for uterine receptivity in animal models (Rhee *et al*., 2016), in immortalized human endometrial stromal cell lines triggered to undergo decidualization (Hill *et al*., 2007; Rhee *et al*., 2016), and in humans as well (Bellver *et al*., 2013). An important factor that would be influencing and playing a key role in endometrial receptivity is leptin (Catteau *et al*., 2016). Human endometrial endothelial cells in culture express the leptin-receptor, through which leptin regulates the remodeling of the human endometrial epithelium (Gonzalez & Leavis, 2001) stimulating proliferation and apoptotic cell pathways in vitro (Tanaka & Umesaki, 2008). In addition, Bellver *et al*. (2011), analyzing gene expression during the window of implantation, showed a different endometrial gene expression among obese patients compared to controls with normal weight, being some of these genes previously related to implantation and unexplained infertility.

In contrast with our results, previous studies involving overweight/obese females that underwent in vitro fertilization with their own oocytes did not demonstrated lower PR, IR, and LBR rates compared to normal-weight females (Vilarino *et al*., 2011; Legge *et al*., 2014; Ozekinci *et al*., 2015; Atzmon *et al*., 2017; Friedler *et al*., 2017; Mackenna *et al*., 2017; Sampo *et al*., 2017; Di Gregorio *et al*., 2019). In these studies, it is important to emphasize that the embryo transfers were made at day 2 or 3 of the development, when the embryonic genome had not yet been activated, and its potential to reach blastocyst stage is unknown, which did not show the effects of overweight and obesity on clinical outcomes. Besides, embryo transfer in earlier developmental stage shows reduced implantation potential when compared with the transfer in blastocyst stage, because the latter enables a better synchronization between the embryo developmental stage and the uterine environment (Quea *et al*., 2007). Furthermore, ethnicity could be playing a possible role on ART results, since in most of these studies did not have Hispanic women among their subjects, and anthropometrics measures can be dramatically different among several ethnicities. In 2011-2013 the prevalence of overweight/obesity in women of reproductive age in Ecuador was 56.8%, with a height average of 152 cm (Freire *et al*., 2013); whereas in the USA it was 38.3% in women of all the races, and 45.7% in Hispanic women during 2011-2014 (Ogden *et al*., 2015). In ART, the prevalence of overweight/obese women that underwent an in-vitro fertilization procedure is variable and dependent on the site where the study was carried out, observing values that oscillate from 28% (Sampo *et al*., 2017) all the way up to 54.4% (Di Gregorio *et al*., 2019), and now 64.9% in our study. These differences correspond to nutritional changes, bad quality and schedules of food, and new lifestyles that lead to an increase in overweight, obesity, and their complications.

Finally, it is important to highlight that the main limitation of our study lies in its retrospective design and the inherent risk for undetected selection bias, and the limited number of cycles. Well-designed prospective studies, in which the variables are controlled and include a greater number of cycles, are needed in order to corroborate the results reported in this retrospective study.

In conclusion, our data shows that overweight and obesity significantly decreases pregnancy, implantational and live birth rates in women undergoing an IVF procedure. Interventions, including weight loss, physical activity, and dietary modification are highly recommended for overweight/obese women wishing to conceive, especially those wishing to have a term liveborn baby.
